# Downregulated Expression of Ly-6-ThB on Developing T Cells Marks CD4^+^CD8^+^ Subset Undergoing Selection in the Thymus

**DOI:** 10.1155/2001/63451

**Published:** 2001

**Authors:** Justin T. Reese, Hitesh Mehta, Clay H. Chappell, Anil. Bamezai

**Affiliations:** Department of Cellular Biology University of Georgia 615 Biological Sciences Bldg Athens GA 30602 USA

**Keywords:** Ly-6, ThB, Thymic selection, TCR signaling

## Abstract

Interaction of TCRs on CD4^+^CD8^+^ immature T cell with MHC-peptide complexes on stromal cells is required for positive and negative selection in the thymus. Identification and characterization of a subpopulation of CD4^+^CD8^+^ thymocytes undergoing selection in the thymus will aid in understanding the mechanisms underlying lineage commitment and thymic selection. Herein, we describe the expression of Ly-6 ThB on developing thymocytes. The majority of CD4^+^CD8^+^ thymocytes express Ly-6 ThB at high levels. Its expression is downregulated in a subset of CD4^+^CD8^+^ thymocytes as well as in mature CD4^+^CD8^-^ and CD4^-^CD8^+^ T cells. More importantly, interaction of TCR/coreceptor with the self-MHC-peptide contributes to the downregulation of ThB expression on developing thymocytes. These findings indicate that downregulation of ThB on CD4^+^CD8^+^ thymocytes identifies a unique subset (CD4+CD8+ThB^neg–low^) of thymocytes that has received the initial signals for thymic selection but have not yet downregulated the CD4 and CD8 cell surface expression. In addition, these results also indicate that a high frequency (Ÿ20–40%) of CD4^+^CD8^+^ immature thymocytes receive these initial signals during thymic selection.


*Developmental Immunology*, 2001, Vol. 8(2), pp. 107-121
Reprints available directly from the publisher
Photocopying permitted by license only

© 2001 OPA (Overseas Publishers Association)
N.V. Published by license under
the Harwood Academic Publishers imprint,
part of The Gordon and Breach Publishing Group.
a member of the Taylor and Francis Group,

### Downregulated Expression of Ly-6-ThB on Developing T Cells Marks CD4$^+$CD8$^+$ Subset Undergoing Selection in the Thymus

JUSTIN T. REESE, HITESH MEHTA, CLAY H. CHAPPELL and ANIL. BAMEZAI*

*Department of Cellular Biology, University of Georgia, Athens, GA 30602, USA*

Interaction of TCRs on CD4$^+$CD8$^+$ immature T cell with MHC-peptide complexes on stromal cells is required for positive and negative selection in the thymus. Identification and characterization of a subpopulation of CD4$^+$CD8$^+$ thymocytes undergoing selection in the thymus will aid in understanding the mechanisms underlying lineage commitment and thymic selection. Herein, we describe the expression of Ly-6 ThB on developing thymocytes. The majority of CD4$^+$CD8$^+$ thymocytes express Ly-6 ThB at high levels. Its expression is downregulated in a subset of CD4$^+$CD8$^+$ thymocytes as well as in mature CD4$^+$CD8$^-$ and CD4$^-$CD8$^+$ T cells. More importantly, interaction of TCR/coreceptor with the self-MHC-peptide contributes to the downregulation of ThB expression on developing thymocytes. These findings indicate that downregulation of ThB on CD4$^+$CD8$^+$ thymocytes identifies a unique subset (CD4$^+$CD8$^+$ThB$^{neg-low}$) of thymocytes that has received the initial signals for thymic selection but have not yet downregulated the CD4 and CD8 cell surface expression. In addition, these results also indicate that a high frequency (~20–40%) of CD4$^+$CD8$^+$ immature thymocytes receive these initial signals during thymic selection.

*Keywords:* Ly-6, ThB, Thymic selection, TCR signaling

### INTRODUCTION

Development of thymocytes and their selection occurs in discreet steps in the thymus. It has become increasingly clear that various cell surface proteins on T lymphocytes interacting with their ligands on the stromal cells are critical for these processes (1). A key set of proteins involved in the thymic development is the TCR/CD3 complex and antigen coreceptors (CD4 and CD8) (2, 3). For example, expression of TCR-beta (pre-TCR complex) is critical for CD4$^-$CD8$^-$ to CD4$^+$CD8$^+$ cell development (2, 3). Expression of TCR-alpha-beta is critical for further development into either CD4$^+$CD8$^-$ helper T cells or CD4$^-$CD8$^+$ cytotoxic T cells (3–5). The underlying mechanisms involved in these developmental proc-

* Corresponding Author: Anil Bamezai, 615 Biological Sciences Bldg, University of Georgia, Athens, GA 30602. Tel. No: 706.542.3607, Fax: 706.542.4271, e-mail: abamezai@cb.uga.edu esses and lineage commitment to $CD4^+$ helper or $CD8^+$ cytotoxic T cell are unclear. The identification and analysis of a subset within $CD4^+CD8^+$ thymocytes that have received initial signals through their TCR's will aid in providing insights into the underlying mechanisms for T cell selection as well as lineage commitment.

ThB is a member of the Ly-6 multigene family expressed on thymocytes, B cells, and keratinocytes (6–9). Similar to other known members of the Ly-6 multigene family, ThB is 10–12 kDa in molecular mass and linked to the cell surface by a GPI-anchor (10, 11). Although the biological function of the ThB molecule is unknown, recent reports indicate that the human homologue of ThB (E48) has cell-cell adhesion function in keratinocytes (9). These latter observations are consistent with the cell adhesion properties reported for other members of the Ly-6 gene family (12–14).

A distinct feature of some Ly-6 proteins is their regulated expression during the development of hematopoietic cells (11, 15). For example, Ly-6A.2 is expressed on stem cells (16, 17) as well as on the a $CD44^+$ subset of $CD4^-CD8^-CD3^-$ T cells in the thymus (18). Ly-6A.2 is absent on the the majority of immature thymocytes including the $CD4^+CD8^+$ subset but reexpressed on mature T cells in the thymus and spleen (19). In this regard, approximately 50–80% of thymocytes express ThB, but expression of this molecule at different developmental stages during thymic development is unknown.

Herein, we present evidence that Ly-6 ThB expression is tightly regulated during thymic development. Interaction of antigen receptor and CD4/CD8 with self-MHC-peptide contributes to the downregulation of ThB on $CD4^+CD8^+$ thymocytes. These results indicate that $ThB^{neg-low}$ $CD4^+CD8^+$ T cells are composed of a subset of thymocytes that have received initial signals through the antigen receptor and coreceptor but have not yet downregulated their coreceptor. In addition, this study also indicates that a high frequency of developing $CD4^+CD8^+$ thymocytes express an antigen receptor that has bias towards reactivity to self-MHC-peptide complexes.

### RESULTS

### Expression of ThB on T cell subsets in the thymus and the periphery

We sought to examine ThB expression in BALB/c mice for each of the four thymic subsets defined by anti-CD4 and anti-CD8 antibodies (Figure 1A). Approximately 40% of the $CD4^-CD8^-$ subset that comprise 2–4% of total thymocytes express high levels of ThB on their surface (DN/R4). A majority of $CD4^+CD8^+$ thymocytes (62%) expresses high levels of ThB (DP/R2). In contrast, 93% of $CD4^+CD8^-$, and 93% of $CD4^-CD8^+$thymocytes are $ThB^{negative-low}$ (Figure 1 A&B). Similar results were obtained with a number of strains of mice including, C57BL/6, SWR, AKR, SJL (data not shown). One notable exception was the higher expression of ThB as well as higher numbers of ThB expressing thymocytes in C57BL/6 mice compared to BALB/c mice, which is consistent with previous report (25). Approximately 80% of the $CD4^+CD8^+$ thymocytes from C57BL/6 mice express higher levels of ThB as compared to about 60% this subset in BALB/c mice. Mature $CD4^+$ and $CD8^+$ T cell subsets in the periphery (lymph node and spleen) do not show surface expression of ThB (data not shown) as previously described (8). These data indicate that ThB expression is downregulated during development of immature $CD4^+CD8^+$ to mature $CD4^+CD8^-$ and $CD4^-CD8^+$ cells. These results also indicate regulated expression of ThB within the $CD4^+CD8^+$ subset of thymocytes and identify novel $ThB^{low}$ and $ThB^{high}$ subpopulations with in $CD4^+CD8^+$ subset.

### ThB surface expression correlates with maturational status of developing T cells in the thymus

As T cells mature in the thymus they lose expression of CD4 or CD8 coreceptors. To evaluate the the expression of ThB during this process we performed three color analysis using anti-CD4, anti-CD8 and anti-ThB flourochrome conjugated antibodies and

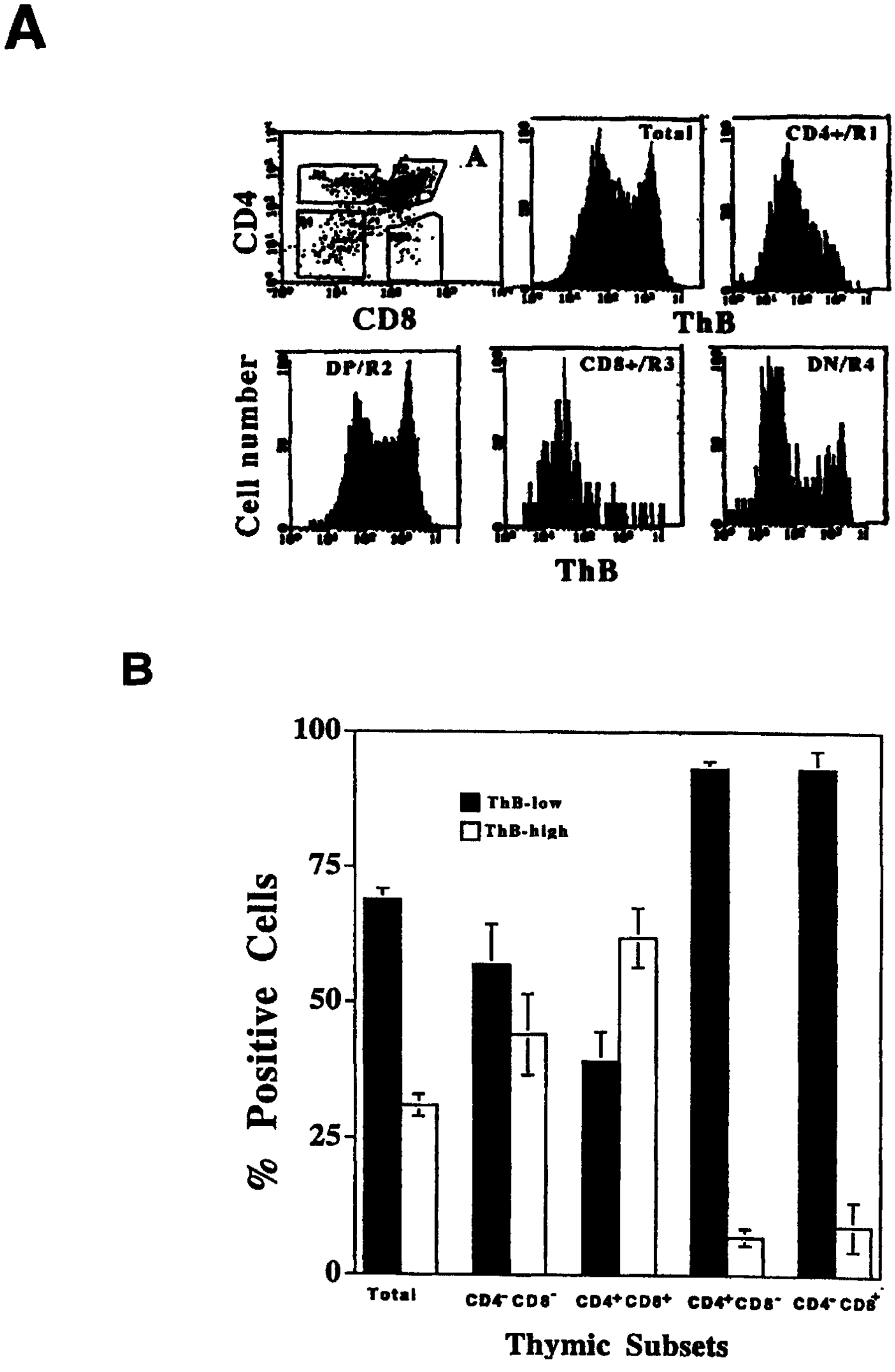

FIGURE 1 Expression of ThB on thymocytes from BALB/c mouse. Thymocytes were isolated and stained with anti-CD4-PE, anti-CD8-FITC, anti-ThB-Biotin and streptavidin-Red as described in Materials and Methods. Each of the four subsets based on the expression of CD4 and CD8 were analyzed for the expression of ThB (A). ThB positive cells in the four thymic subsets, $CD4^-CD8^-$, $CD4^+CD8^+$, $CD4^+CD8^-$ and $CD4^-CD8^+$ from six independent experiments are shown (B) (n=6). Error bars indicate standard error gated on either DP thymocytes or $CD4^+$ thymocytes that have partially or completely downregulated the CD8 coreceptor. Figure 2 shows that the expression of ThB is lowest in $CD4^+CD8^-$ T cells and intermediate in $CD4^+CD8^{low}$ and highest in $CD4^+CD8^+$ T cells in the thymus. Similarly $CD4^{low}CD8^+$ T cells also expressed lower levels of ThB (Figure 2) and $CD4^-CD8^+$ showed the lowest expression like $CD4^+CD8^-$ helper subset (data not shown). These results strongly suggest that as thymocytes mature and lose CD4 or CD8 coreceptors, there is a coordinate loss of ThB.

Maturation of $CD4^+$ helper and $CD8^+$ cytotoxic T cells from $CD4^+CD8^+$ immature T cell involves upregulation of TCR/CD3, CD5 proteins and downmodulation of CD4 or CD8 molecules. To further examine the expression of ThB on developing T cells we analyzed BALB/c thymocytes using anti-ThB and anti-CD5 (Figure 3 A&B). Majority of the thymocytes that show higher expression of CD5 have downregulated ThB expression (65.1+/-8%). A subset of thymocytes (28.3+/-12%) with higher expression of CD5 also have high expression of ThB. These suggest that with in $CD5^+$ thymocytes both $ThB^{high}$ and $ThB^{low}$ subpopulations exist. Similar analysis using anti-ThB and anti-CD3 antibodies indicated that majority of $CD3^{high}$ (approximately 16.5%) (mature subset) expressed lower levels of ThB. Interestingly, about 25% of $CD3^{neg\text{-}intermidiate}$ (immature subset) cells show downregulation of ThB. This analysis indicates that as T cells become mature (i.e., upregulation of CD5 and TCR) they lose ThB expression from the cell surface. Taken together our data strongly suggests that downregulation of ThB expression correlates with the maturity of developing T cells in the thymus.

### Regulation of ThB surface expression by interaction of antigen receptor and co-receptor with self-MHC-peptide complexes

The progression of $CD4^+CD8^+$ thymocytes to the mature $CD4^+CD8^-$ or $CD4^-CD8^+$ thymocytes require ligation of the TCR and the CD4 or CD8 coreceptor. We hypothesized that interaction of the antigen receptor with self-MHC results in the downregulation of ThB expression on $CD4^+CD8^+$ thymocytes. To examine this hypothesis we determined the expression of ThB in the AND TCR transgenic mice that express the transgene either on a selecting ($H\text{-}2^b$, B10) or non-selecting ($H\text{-}2^d$ B10.D2) background (21). The expression of ThB was lower on thymocytes from AND-$H\text{-}2^b$ mice than on the T cells from AND-$H\text{-}2^d$ mice (Figure 4A). As reported previously, we also observed a higher percentage of $CD4^+$ cells in AND transgenic mice on $H\text{-}2^b$(Selecting) as compared to $H\text{-}2^d$ (non-Selecting) background (21). Further analysis of the $ThB^{neg}$, $ThB^{low}$ and $ThB^{high}$ subsets in AND transgenic mice on selecting and non-selecting genetic backgrounds indicated that almost all the $ThB^{neg}$ thymocytes are either $CD4^+$ mature or $CD4^-CD8^-$ immature T cells (Figure 4B). In contrast, the majority of $ThB^{high}$ thymocytes belonged to the $CD4^+CD8^+$ subset. Interestingly, the $ThB^{low}$ thymocytes included both immature $CD4^+CD8^+$ thymocytes as well as mature $CD4^+$and $CD8^+$ T cells. Moreover, a higher percentage of $ThB^{high}$ cells were observed in AND transgenic mice under non-selecting (24%) than the selecting (9.7%) background. This was consistent with observation that higher percentages of $ThB^{low}$ T cells were observed under selecting (60%) than non-selecting (49%) backgrounds. In addition a majority of T cells maturing under "non-selecting" ($RAG^+$) backgound also downregulate the expression of ThB and therefore indicate that non-selecting background does provide weak positive slection. These findings suggest that the expression of ThB correlates with the positive selection of T cells in TCR transgenic mice and these results are consistent with what we observe in normal non-transgenic mice (Figure 1).

We have extended our studies using several TCR transgenic mice: (1) 5C.C7 TCR transgene (22) expressed on selecting (B10) and non-selecting (B10.A) genetic backgrounds (Figure 5). 2. DO11 TCR transgenic that recognizes the c-ovalbumin peptide in the context of the MHC class II molecule $I\text{-}A^d$ (20). 3. H-Y TCR transgenic (23) female B10 mice (data not shown). $CD4^+$ T cells selected in th DO11 TCR transgenic mice and $CD8^+$ T cells selected in the H-Y TCR transgenic female mice have down regulated expression of ThB (data not shown). Taken

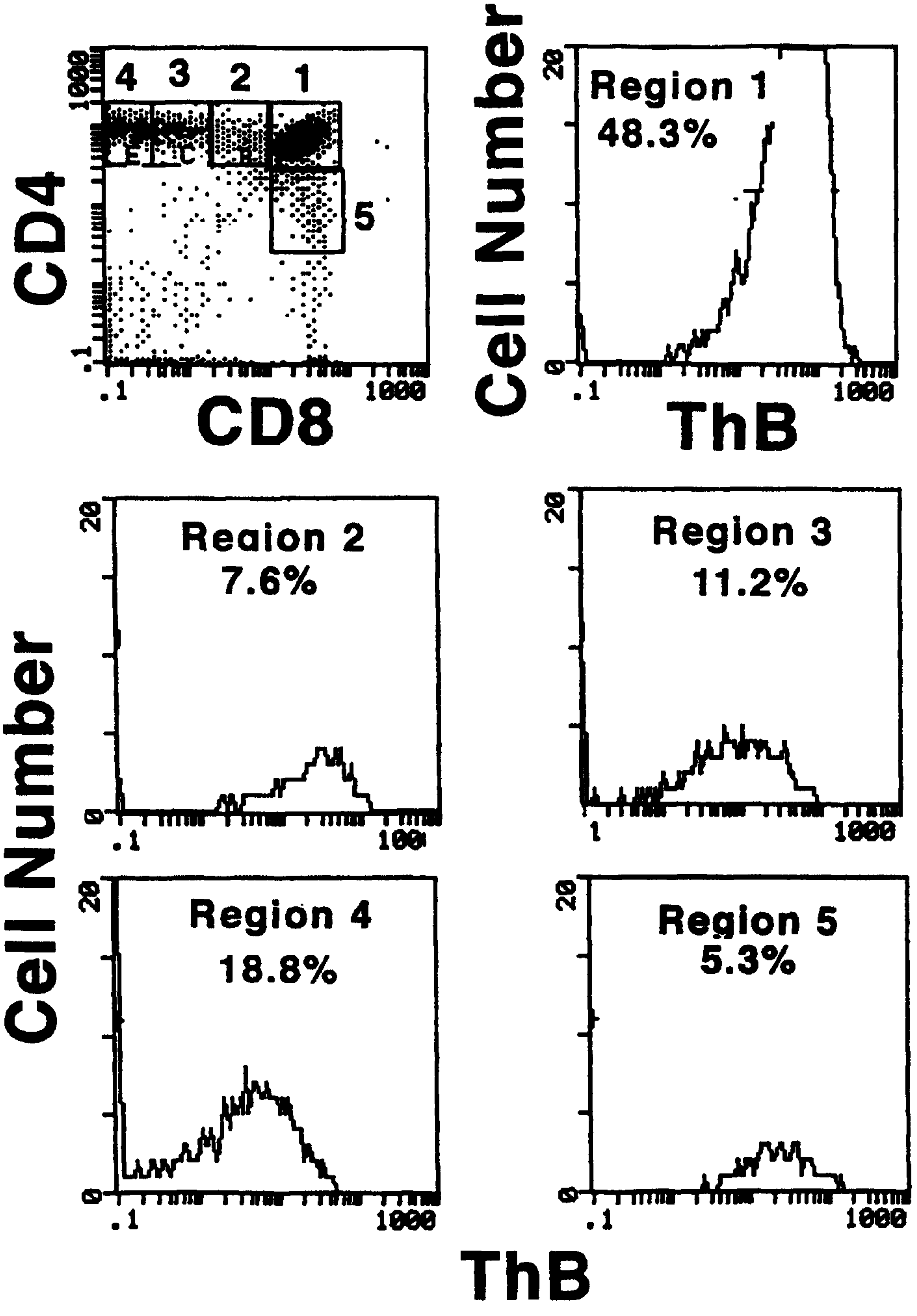

FIGURE 2 Expression of ThB on thymocytes with variable expression of the CD4 coreceptor. Thymocytes were isolated and stained as in Figure 1 and as described in Materials and Methods. Regions 1–5 were drawn based on expression of the coreceptors CD4 and CD8 indicated on each panel. Expression of ThB on thymocytes in each region is shown. Numbers at the top of each panel represents percentage of total thymocyes in the region drawn. A representative experiment of three different analyses is shown

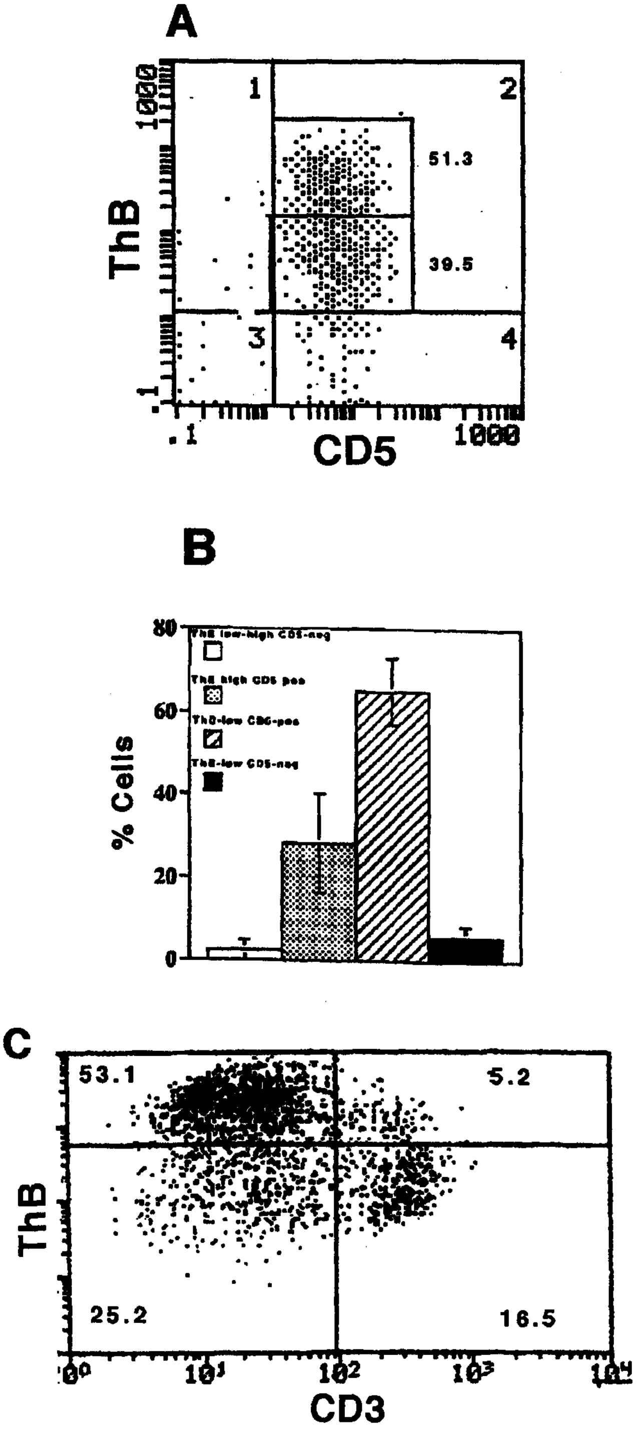

FIGURE 3 Expression of CD3, CD5 and ThB on thymocytes. BALB/c thymocytes were stained with anti-ThB-Biotin and either anti-CD5-FITC (A) or anti-CD3-FITC (C), followed by strepavidin-PE. Panel B shows mean percentages +/-S.D of cells expressing CD5 and ThB molecule (n=4 experiments)

together, our data strongly indicates that interaction of TCR and CD4 or CD8 coreceptor with self-MHC down regulates expression of ThB.

### Expression of ThB in MHC class I and II negative thymocytes

If the TCR/coreceptor – MHC interaction is indeed down regulating the expression of the ThB molecule, then preventing these interactions should also prevent ThB down regulation. To test this hypothesis, ThB expression on thymocytes from MHC class I°II° mice was examined. In these mice there can be no interaction of TCRs and CD4 on developing T cells due to the lack of expression of MHC-peptide complexes. Therefore, developing T cells do not get an increased ThB expression compared with wild type C57Bl/6. These observations further substantiate the hypothesis that the interaction of TCR/coreceptor with self-MHC-peptide complexes is necessary to down-regulate the cell surface expression of ThB on $CD4^+CD8^+$ thymocytes.

### Downregulation of ThB expression on thymocytes from MHC class $I^-II^-$ mice injected with anti-CD3 and anti-CD4 antibodies

Our data indicates that engagement of the TCR/coreceptor is necessary for the down regulation of ThB expession. To determine if the ThB expression can be experimently downregulated by engagement of both the TCR and the coreceptor we injected anti-CD3 along with anti-CD4 antibodies in mice that lack the expression of MHC class I and II molecules. Thymocytes from the MHC class $I^-II^-$ mice are blocked in their development at the $CD4^+CD8^+$ cell stage and the majority show high cell surface expression of ThB. Analysis of the thymus on day 6 post injection revealed the presence of a subset of cells with low ThB (Figure 7A). The downregulation of ThB was specific because expression of HSA was not altered in these mice (Figure 7B). This expression is not altered in MHC-deficeint mice injected with saline (controls) (Figure 7A) as well as anti-CD4 + isotype control antibody (data not shown). Moreover the downmodulation of ThB was not observed on day 2 after the injection of anti-CD3 + anti-CD4 antibody (data not shown). Injection of antibody did not alter the cell numbers recovered from the thymus ($8.6x10^7$ in saline injected Vs $1x10^8$in anti-CD3+anti-CD4 injected mice), therefore indicating that anti-CD3 and anti-CD4 antibodies did not induce cell death in ThB-$^{high}$ subset (Figure 7A). These results suggests that ligation of TCR and CD4 proteins is required for downregulation of ThB expression.

### DISCUSSION

An interesting feature of the products of the Ly-6 locus is their regulated expression during development (11, 26). Ly-6A.2, one of the better-studied members of this gene family, shows tightly regulated expression during thymic development. Dysregulating the expression of Ly-6A.2 on T cells in the thymus induces a block in development at the $CD4^-CD8^-$ cell stage, where normal expression of Ly-6A.2 is lost (18). These observations indicate that regulated expression of Ly-6A.2 is important for normal T cell development (18). The data in this report indicates that ThB also shows regulated expression on developing T cells in the thymus that is distinct and regulated at different stages of thymic development than Ly-6A.2. High ThB expression is observed on some $CD4^-CD8^-$, on the majority of $CD4^+CD8^+$ thymocytes and its expression is lost on mature $CD4^+CD8^-$ and $CD4^-CD8^+$ T cells. Similarly, lower expression of ThB also correlates with the maturity of developing T cells in the thymus as defined by CD3 and CD5 expression. Interestingly, the expression of CD5 on $CD4^+CD8^+$ thymocytes is not biphasic (27) where as expression of ThB does dissect CD4+CD8+ subset into two subsets (Figure 1). These results indicate that ThB is a unique cell surface marker on developing T cells whose expression further dissects the CD5 expressing developing T cells in the thymus.

Study of the expression of CD44 and CD25 has defined stages in the development of the $CD4^-CD8^-$ (DN) subset of thymocytes. Unlike the DN subset, markers for the progression of development within the DP subset are limited. Previous studies have sug-

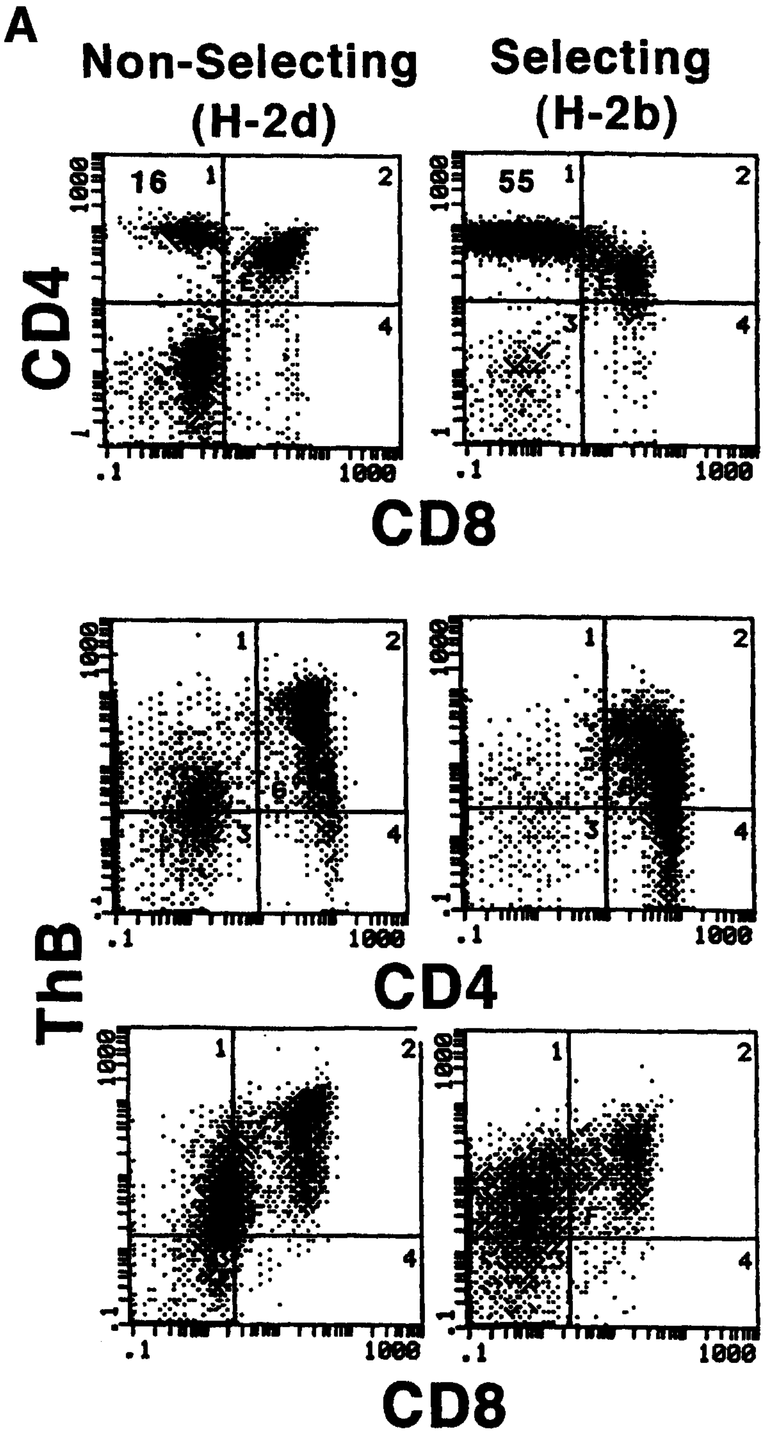

A

Non-Selecting (H-2d)

Selecting (H-2b)

CD4

CD8

ThB

CD4

CD8

16

55

.1

1000

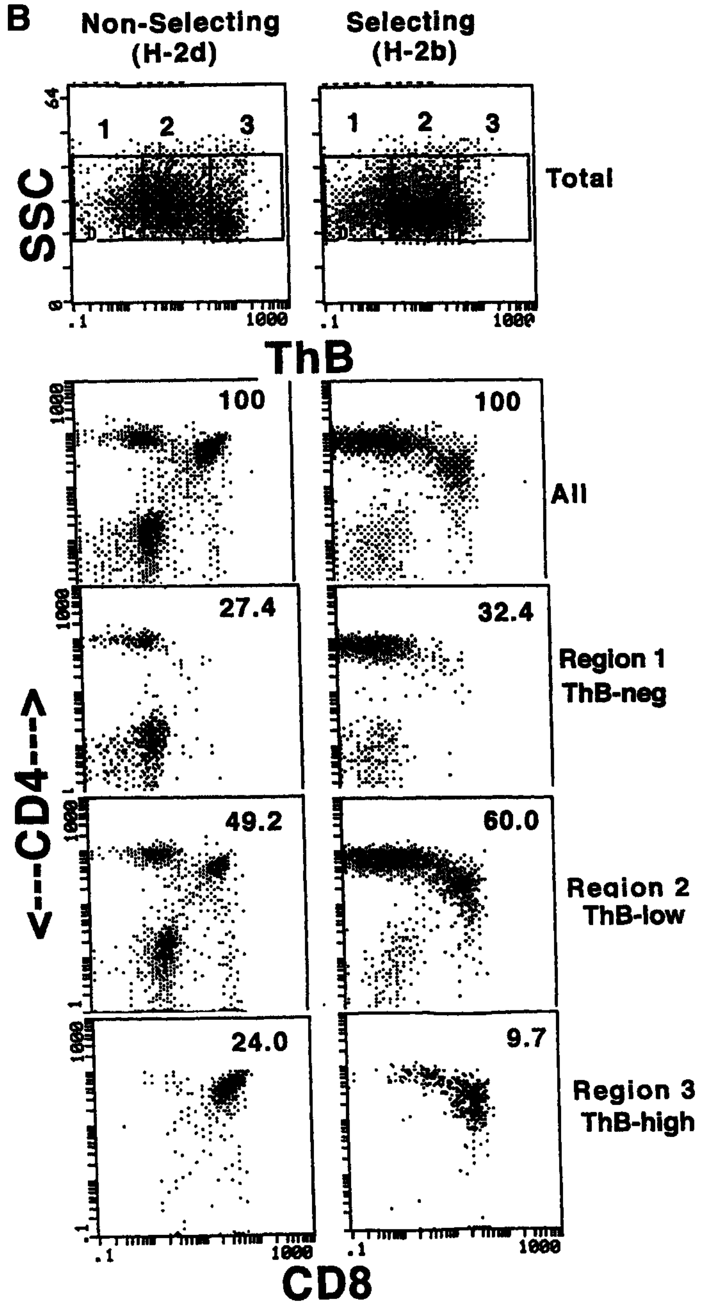

FIGURE 4 Expression of ThB on AND TCR transgenic thymocytes. Thymocytes were isolated and stained with anti-CD4-PE, anti-CD8-FITC, anti-ThB-Biotin followed by streptavidin-Red as described in Materials and Methods. (A) CD4 and CD8 expression on total thymocytes (gated by forward and side scatter) are shown (Upper two panels). Number in the upper left quadrant represents indicate percentages of the thymic subset. Expression of ThB and CD4 (Middle two panels) and ThB and CD8 (lower two panels) is shown. (B) Analysis of ThBhigh, ThBlow and ThBneg expression on thymocytes from AND TCR transgenic mice. Thymocytes were stained in (A) with anti-CD4-PE, anti-CD8-FITC and anti-ThB-Biotin followed by strepavidin-red 670 were regioned based on the expression of ThB (upper panel). ThBhigh, ThBlow and ThBneg thymocytes were analysed for the expression of CD4 and CD8 molecules (middle and lower panels). All data are representative of two experiments

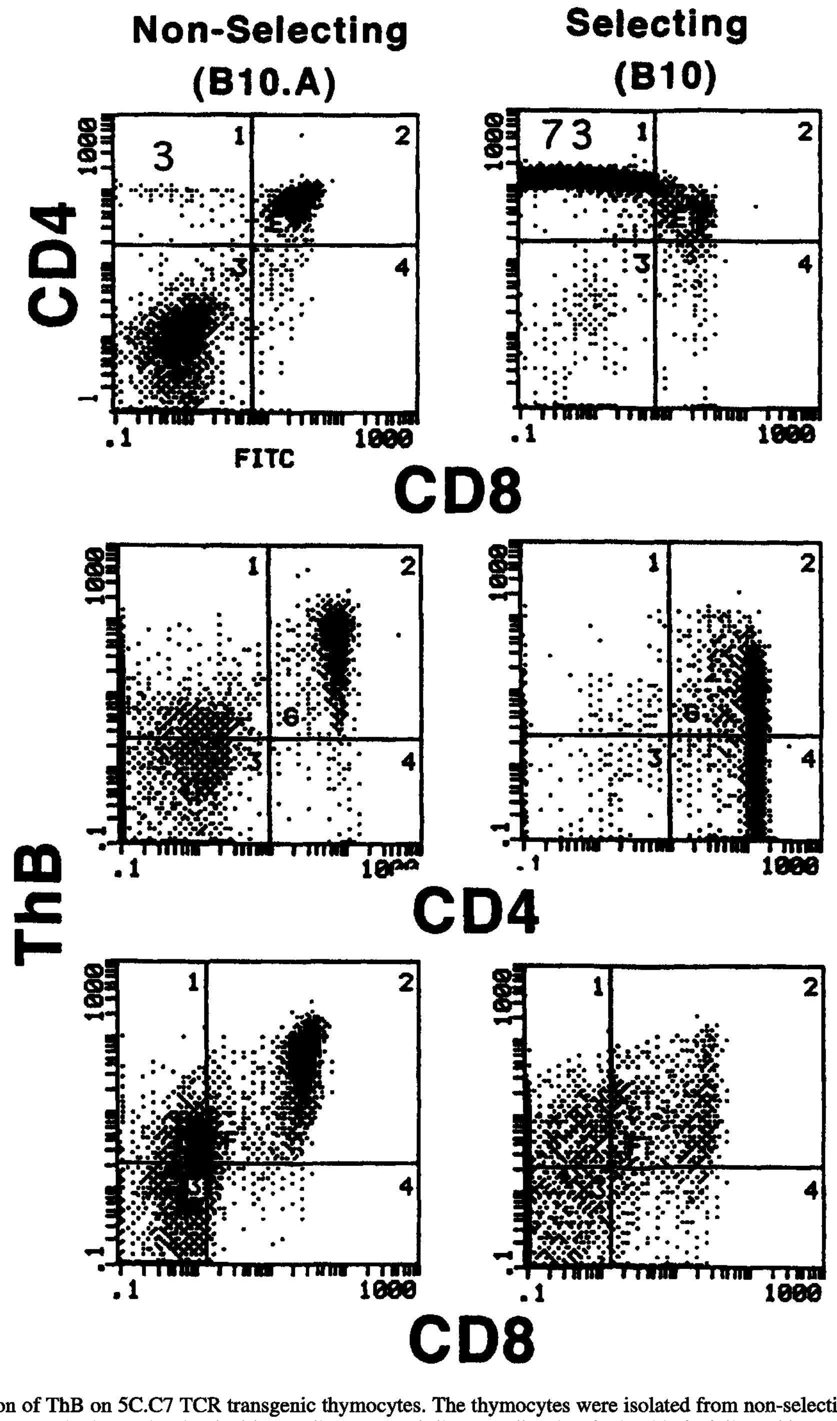

FIGURE 5 Expression of ThB on 5C.C7 TCR transgenic thymocytes. The thymocytes were isolated from non-selective (B10.A) and selective (B10) MHC background mice and stained with anti-CD4-PE, anti-CD8-FITC and anti-ThB-biotin followed by strepavidin-red670. CD4 and CD8 expression on total thymocytes (gated by forward and side scatter) are shown (Upper two panels). Number in the upper left quadrant represents indicate percentages of the thymic subset. Expression of ThB and CD4 (Middle two panels) and ThB and CD8 (lower two panels) is shown. All data are representative of two experiments gested that the expression of CD3, HSA and CD69 can define the differentiation stage of thymocytes within the DP subset. DP $CD3^{hi}$ thymocytes are thought to arise from DP $CD3^{int}$ thymocytes (28). HSA is highly expressed on the majority of DP thymocytes and is down regulated in the majority of mature T cells in the medulla (29). Moreover, up regulation of CD69 seems to correlate with the maturation of DP thymocytes to the SP cell stage (30). Only 10% of $CD4^+CD8^+$ thymocytes upregulate CD69 and audition for T-cell antigen receptor mediated positive selection (30, 31). The level of ThB can be used to further dissect this subset of thymocytes. Therefore ThB expression defines unique subset with in the T cell subset ($CD4^+CD8^+$) in the thymus of normal mice known to undergo thymic selection. Isolation and characterization of this subset will provide further insights into T cell development in the thymus.

Although it is noteworthy that the expression of a number of Ly-6 molecules fluctuate during T cell development in the thymus, how this regulation occurs is unknown. Here we describe, that one factor which contributes to the regulation of Ly-6 ThB is interaction of TCR/coreceptor with self MHC + peptide. The experimental evidence supporting this conclusion are: First, lower expression of ThB expression on thymocytes from TCR transgenic mice expressed on selecting than the non-selecting MHC background. This decrease in ThB expression was observed on $CD4^+CD8^+$ thymic subset. Second, in MHC mutant mice, in which MHC interactions with TCR are essentially absent, ThB expression remained high on thymocytes. 3. Injection of a combination of anti-CD3 and anti-CD4 antibodies (Figure 7) in MHC-deficient mice resulted in downmodulation of ThB on thymocytes. 4. Endogenous expression of ThB in wild type mice correlates with the maturation of developing T cells in the thymus. These results suggest that antigen receptor and coreceptor ligation is necessary for downmodulation of ThB. It is possible that additional proteins expressed on $CD4^+CD8^+$ T cells including ThB as well as other cell-autonomous mechanisms may also contribute to the downmodulation of ThB.

Previous studies indicate that addition of anti-CD3 and anti-CD4 antibodies to fetal thymi from MHC class $I^-II^-$ mice results in maturation of $CD4^+$ T cells and downregulates the expression of HSA from $HSA^{high}$ to $HSA^{low}$ therefore suggesting that these cells have undergone positive selection. In contrast, our studies in which anti-CD3 and anti-CD4 antibodies are injected in vivo does not result in maturation of $CD4^+$ helper T cells. Moreover, the expression of HSA also remains unaltered in these injected mice. These different results may be attributed to the different experimental systems used (i.e. fetal thymic organ culture Vs injection into the mice) or differences in the amount of antibodies reaching thymocytes under these different experimental conditions. Interestingly, injection of anti-CD3 and anti-CD4 antibodies in MHC class $I^-II^-$ mice cause downregulation of ThB expression on $CD4^+CD8^+$ thymocytes. These later observations may suggest that anti-CD3+anti-CD4 antibodies do not provide sufficient ligation for full positive selection to proceed but may initiate some initial events in this process (i.e. downregulation of ThB expression). These later results are consistent with other data that downregulation of ThB requires interaction of TCR and co-receptor with their ligand.

Previously published data have demonstrated two patterns of ThB expression (25). Thymocytes from C57BL/6 mice show higher ThB expression, in comparison with thymocytes from BALB/c mice which are known to show lower levels of ThB. Our observations are consistent with these published results since only about 80% of $CD4^+CD8^+$ thymocytes express high levels of ThB in C57BL/6 mice as compared to 50–60% in BALB/c mouse. These differences do not arise from expression of different alleles in these two mouse strains (32). The genetic basis of these differences is not known. The genetic variation of ThB expression has not been mapped to H-2 locus and therefore lack of I-E expression in C57BL/6 is unlikely to influence ThB expression (6). These results suggest that ligation of TCR and CD4 molecules are one of many factors that may contribute to downregulation of ThB expression. Contribution of

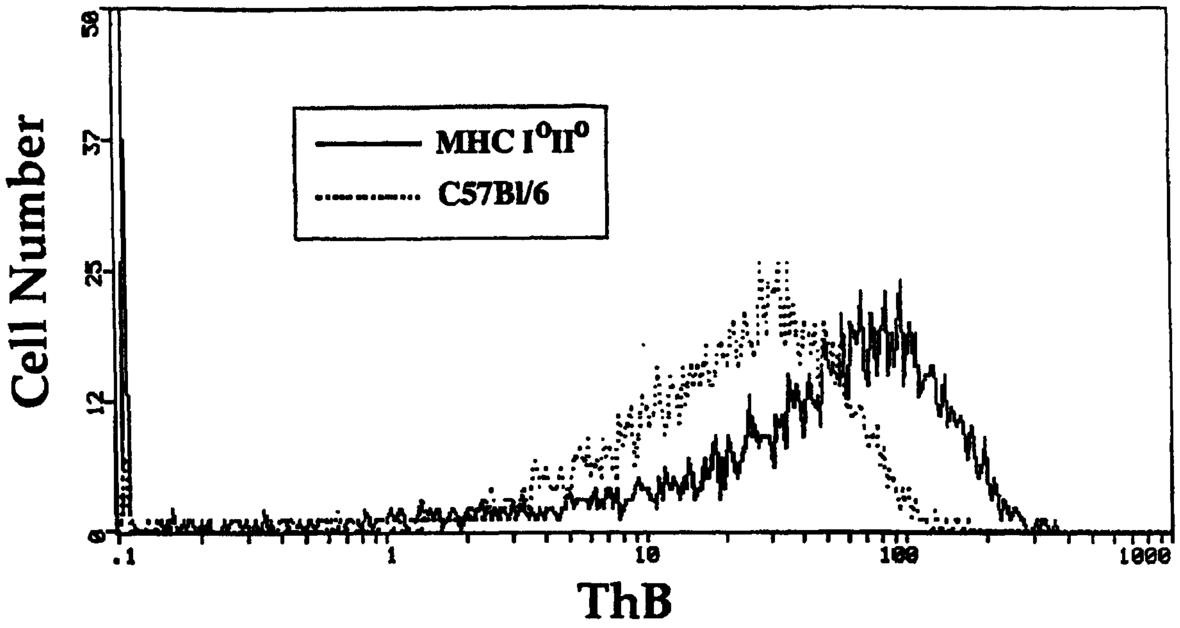

FIGURE 6 Expression of ThB on MHC class I$^-$II$^-$thymocytes. Thymocytes were isolated and stained with anti-CD4-PE, anti-CD8-FITC and anti-ThB-Biotin followed by streptavidin-Red as described in Materials and Methods. ThB expression on double positive (DP) thymocytes was determined by gating on DP thymocytes and measuring streptavidin-Red fluorescence. These data are representative of three experiments additional cell surface proteins is possible but yet undetermined.

The $CD4^+CD8^+$ subset of thymocytes represents a critical stage where MHC restriction, lineage commitment and thymic selection occur. The underlying mechanisms for these processes are largely unknown. Identification of the transition phases within the $CD4^+CD8^+$ subset that are dependent on the interaction of TCR/coreceptor with MHC may aid in the isolation and analysis of cells auditioning for thymic selection. Our results also indicate that this interaction regulates the expression of ThB at the DP cell stage and we are able to identify a unique $CD4^+CD8^+ThB^{low}$ subset in the thymus. The $CD4^+CD8^+ThB^{low}$ cells constitute about 20–40% of the total thymocyte pool. Based on the data from non-transgenic, TCR-transgenic and MHC null mice, $CD4^+CD8^+ThB^{low}$ cells seems to have received initial signals of thymic selection. Our results are unable to determine whether $CD4^+CD8^+$ thymocytes down-regulating expression of Ly-6-ThB are undergoing or destined to be negatively or positively selected. Given previous reports that all the superantigen-reactive TCR-Vß bearing T cells are included and present in the $CD4^+CD8^+$ subset but absent in the mature $CD4^+$ or $CD8^+$ subsets (33) suggests that downmodulation of ThB occurs prior to disappearance of cells by negative selection and may be the result of first and initial interaction of TCR/coreceptor with self-MHC+peptide as previously proposed (34).

This data also suggests that a high percentage of DP thymocytes that have rearranged their TCR react with self-MHC-peptide complexes that results in downmodulation of ThB expression. These results are consistent with other recent observations that T cell repertoire that exists prior to thymic selection is highly biased in its reactivity to MHC molecules (35–37). The data presented in here are consistent with our previous estimates that indicate that this reactivity is atleast in the range of 10–15% (35). In addition,

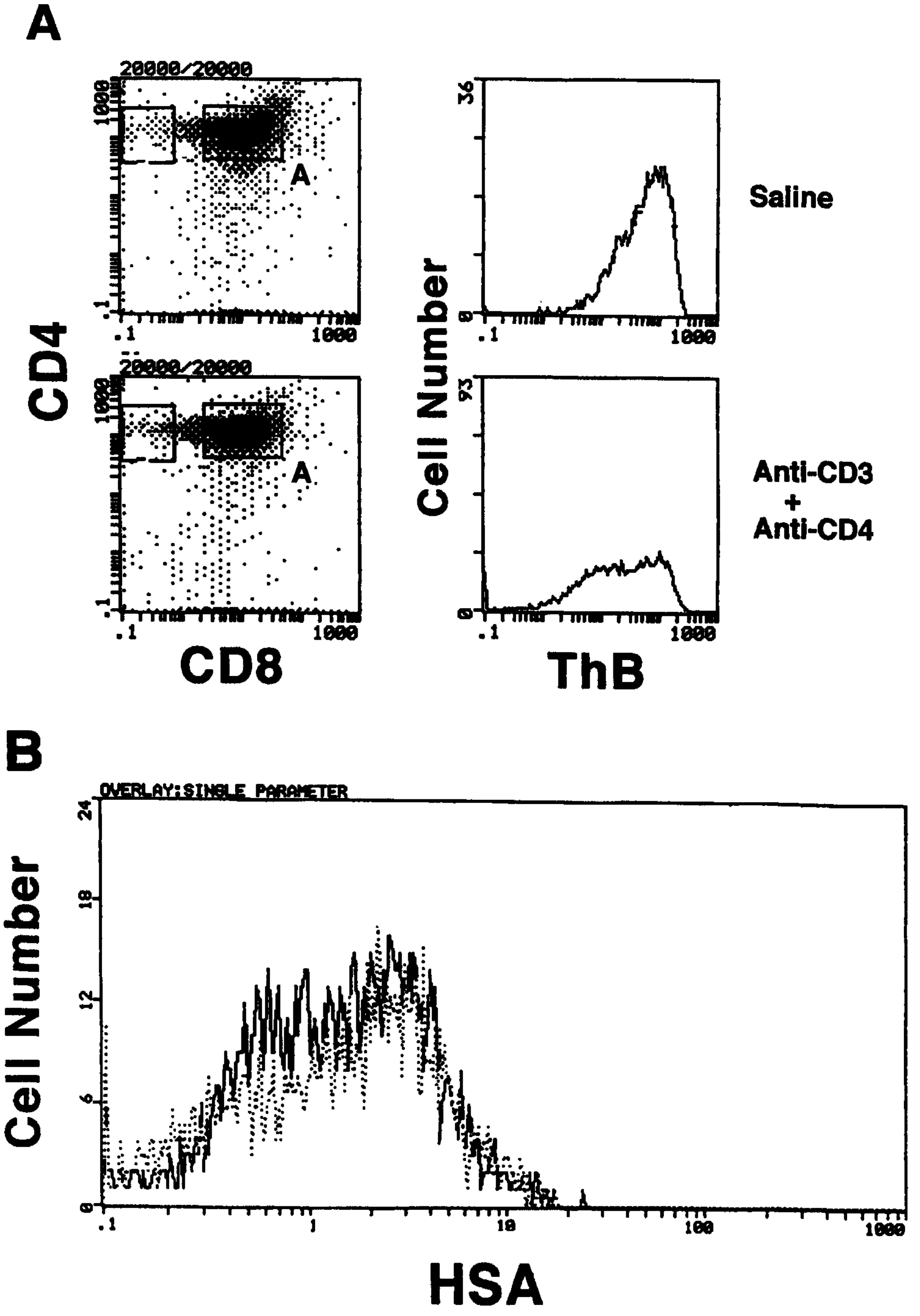

FIGURE 7 Effect of anti-CD3 and anti-CD4 mAb's on ThB and HSA expression in vivo. MHC class I⁻II⁻ mice were injected with either saline or anti-CD3 (145–2C11) + anti-CD4 (GK1.5). Thymocytes from injected mice were stained six days post injection with anti-CD4-PE, anti-CD8-FITC and anti-ThB-Biotin followed by strepavidin-red670. CD4+CD8+ T cells were gated and analyzed for the expression of ThB (A). Expression of HSA was also analyzed on the thymocytes from saline (solid line) and anti-CD3 + anti-CD4 (dotted line) injected mice

TCR-alpha gene loci are reported to undergo secondary gene rearrangement that would increase the possibility of generating TCRs with reactivity to self-MHC and peptide complexes (38).

The biological significance of downregulation of ThB is unclear. Considering the expression pattern of ThB (this manuscript) and adhesion properties of human homologue of ThB (E48) (9) as well as other Ly-6 proteins (12–14), we hypothesize that one of the functions of ThB is to hold thymocytes in the appropriate thymic region where thymic selection occurs. Therefore, downregulation of ThB will aid in de-adhesion and movement of maturing T cells towards the medulla. Alternatively it is also possible that ThB participates in positive or negative selection which might alter signaling threshold on T cells undergoing selection. This possibility is consistent with signaling property of Ly-6 proteins (11). The generation of ThB transgenic mice and null mutants will provide better insight into this phenomenon.

### MATERIALS AND METHODS

### Mice

BALB/c, C57B1/6, AKR, SWR, SJL, DO.11 TCR transgenic (20) (generous gift from Dr. Dennis Lo), AND TCR transgenic (21), 5C.C7 TCR transgenic (22) and H-Y transgenic (23) (generously provided by Dr. Polly Matzinger), MHC class $I^-$ and $II^-$ mice (4) (generously provided by Dr. Lauri Glimcher) were used in this study. Mice were bred at the University of Georgia animal facility and used between the ages of 4–10 weeks for the experiments.

### Cell Preparations and Immunofluorescence

Cell were prepared from lymphoid organs (thymus, spleen) and stained with fluorochrome conjugated antibodies according to previously published protocols (24). Briefly, cells were stained with conjugated antibodies, phycoerythrin (PE) – anti-CD4 antibody (H129.19), fluoroscein isothiocyanate (FITC) – anti-CD8, biotin – anti-ThB (49-H4) followed by Streptavidin -Red 613™ (Gibco-BRL). In some experiments thymocytes and splenocytes were stained with 145–2C11 (anti-CD3), 53.9.2 (anti-ThB) followed by goat anti-rat IgG-FITC. Cells were fixed with paraformaldehyde (1% final concentration) before analysis by cytoflourometery (Coulter Epics Elite).

### In vivo modulation of ThB expression

Purified anti-CD3 (145–2C11), anti-CD4 (GK1.5), Hamster IgG (9-A2A4) (50 ug/each) or saline was injected intraperitoneally in 4–8 week old MHC class I-II- mice in a volume of 100 ul. The mice were sacrified 2–6 days post injection and cellularity of the thymus was determined followed by analysis by immunofluorescence and flow cytometery.

*Acknowledgements*

Authors thank Dr. Ken Rock for reagents and cell lines, Dr. Polly Metzinger for making some TCR transgenic mice available for experiments and Drs. Kenneth Rock, Rick Tarleton and Hung-Sia Teh for critically reading the manuscript. This work was supported by the University of Georgia Research Foundation (AB).

***References***

1. Robey, E., Fowlkes, B. J., Selective events in T cell development. (1994) *Annu Rev Immunol.*, **12**: 675–705.
2. Philpott, K., Viney, J., Kay, G., Rastan, S., Gardiner, E., Chae, S., Hayday, A. & Owen, M., Lymphoid Development in Mice Congenitally Lacking T Cell Receptor αβ- Expressing Cells. (1992) *Science*. **256**: 1448–52.
3. Mombaerts, P., Clarke, A., Rudnicki, M., Iacomini, J., Itohara, S., Lafaille, J., Wang, L., Ichikawa, Y., Jaenisch, R., Hooper, M. & Tonegawa, S. (1992) Mutations in T-cell antigen receptor genes α and β block thymocyte development at different stages. *Nature*. **360**: 225–31.
4. Grusby, M. J., Johnson, R. S., Papaioannou, V. E., and Glimcher, L. H. (1991) Depletion of $CD4^+$ T cells in major histocompatibility complex class II-deficient mice. *Science*. **253**: 1417–20.
5. Gosgrove, D., Gray, D., Dierich, A., Kaufman, J., Lemerur, M., Benoist, C., and Mathis, D. (1991) Mice lacking MHC class II molecules. *Cell*. **66**: 1051–66.
6. Eckhardt, L. & Herzenberg, L. (1980) Monoclonal antibodies to ThB detect close linkage of Ly-6 and a gene regulating ThB expression. *Immunogenetics*. **11**: 275–91.

7. Matossian-Rogers A., Rogers, P., Ledbetter, J.A., Herzenberg LA Matossian-Rogers, Roger, Ledbetter, J, Beatty, Hanson. (1982) Molecular weight determination of two genetically linked cell surface murine antigens: ThB and Ly-6. *Immunogenetics.* **15**: 591–9.

8. Ledbetter, J. & Herzenberg L. (1979) Xenogeneic monoclonal antibodies to mouse lymphoid differentiation antigens. *Immunol Rev.* **47**: 363–90.

9. Brakenhoff, R., Gerretsen, M., Knippels, E., Van Dijk, M., Van Essen, H., Weghuis, D., Sinke, R., Snow, G. & Van Dongen, G. (1995) The Human E48 Antigen, Highly Homologous to the Murine Ly-6 Antigen ThB, is a GPI-anchored Molecule Apparently Involved in Keratinocyte Cell-Cell Adhesion. *J. Cell Biol.* **129**: 1677–89.

10. Gumley, T., McKenzie, I. & Sandrin, M. (1995) Tissue expression, structure and function of the murine Ly-6 family of molecules. *Immunol. Cell Biol.* **73**: 277–96.

11. Rock, K., Reiser, H., Bamezai, A., McGrew, J. & Benacerraf, B. (1989) The Ly-6 Locus: A Multigene Family Encoding Phosphatidylinositol-Anchored Membrane Proteins Concerned with T-cell Activation. *Immunol. Rev.* **111**: 195–224.

12. Bamezai, A. & Rock, K. (1995) Overexpressed Ly-6A.2 mediates cell-cell adhesion by binding a ligand expressed on lymphoid cells. *Proc. Natl. Acad. Sci.* **92**: 429498.

13. Johnson, R., Lancki, D.W., and Fitch, F.W. (1993) Acessory molecules involved in antigen mediated cytolysis and lymphokine production by cytotoxic T lymphocyte subset. *J. Immunol.* **151**: 2986–99.

14. Hanninen, A., Jaakkolla, I., Salmi, M., Simell, O., and Jalkanen, S. (1997) Ly-6C regulates endothelial adhesion and homing of $CD8^+$ T cells by activating integrin-dependent adhesion pathway. *Proc. Natl. Acad. Sci. USA.* **94**: 6898–903.

15. Shevach, E., Korty, P. (1989) Ly-6: A Multigene Family in Search of a Function. *Immun. Today.* **10**: 195–200.

16. Yeh, E. T. H., Reiser, H., Benacerraf, B., and Rock, K. L. (1986) The expression, function and ontogeny of a novel T cell-activating protein, TAP, in the thymus. *J. Immunol.* **137**: 1232–8.

17. Spangrude, G. J., Heimfeld, S., and Weissman, I. L. (1988) Isolation and characterization of the mouse hematopoietic stem cell. *Science.* **241**: 58–62.

18. Bamezai, A., Palliser, D., Berezovskaya, A., McGrew, J., Higgins, K., Lacy, E. & Rock, K. (1995) Regulated expression of Ly-6A.2 is important for T cell development. *J. Immunol.* **154**: 4233–39.

19. Yeh E.T, Reiser H., Daley J., Rock K.L. (1987) Stimulation of T cells via the TAP molecule, a member in a family of activating proteins encoded in the Ly-6 locus. *J. Immunol* **138**: 91–7.

20. Murphy, K., Heimberger, A., Loh, D. (1990) Induction by antigen of intrathymic apoptosis of $CD4^+CD8^+TCR^{lo}$ thymocytes in vivo. *Science*, **250**: 1720–3.

21. Kaye, J., Vasquex, N. J., and Hedrick, S. M. (1992) Involvement of the same region of the T cell antigen receptor in thymic selection and foreign peptide recognition. *J. Immunol.* **148**: 3342–53.

22. Berg, L.J., Fazekas de St. Groth, F., Ivars, C., Goodnow, C., Gilfillan, S., Garchon, H.J., and Davis, M. (1988) Expression of T cell receptor alpha chain genes in transgenic mice. *Mol. Cell. Biol.* **8**: 5459–69.

23. Kisielow, P., Bluthmann, H., Staerz, U., Steinmetz, M. and von Boehmer, H. (1992) Tolerance in T-cell-receptor transgenic mice involves deletion of nonmature $CD4^+8^+$ thymocytes. *Nature.* **333**: 742–6.

24. Bamezai, A., Reiser, H. & Rock, K. (1988) T Cell Receptor/CD3 Negative Variants Are Unresponsive To Stimulation Through The Ly-6 Encoded Molecule, TAP. *J. Immunol.* **141**: 1423–28.

25. Gumley, T., McKenzie, I. & Sandrin, M. (1994) Polymorphism at the ThB locus. *Immunogenetics.* **39**: 390–4.

26. Godfrey, D., Masciantonio, M., Tucek, C., Malin, M., Boyd, R. & Hugo, P. (1992) Thymic Shared Antigen-1: A Novel Thymocyte Marker Discriminating Immature from Mature Thymocyte Subset. *J. Immunol.* **148**: 2006–11.

27. Vacchio M.S., Lee J.Y., Ashwell J.D. (1999) Thymus-derived glucocorticoids set the thresholds for thymocyte selection by inhibiting TCR-mediated thymocyte activation. *J. Immunol* 1999. **163**: 1327–33.

28. Lucas B., and Germain R. (1996) Unexpectedly complex regulation of CD4/CD8 coreceptor expression supports a revised model of $CD4^+CD8^+$ thymocyte differentiation. *Immunity.* **5**: 461–77.

29. Bruce, J., Symington, F.W., McKearn, T.J., and Sprent, J. (1981) A monoclonal antibody discriminating subsets of T and B cells. *J. Immunol.* **127**: 2496–501.

30. Yamashita, I., Nagata, T., Tada, T. & Nakayama, T. (1993) CD69 cell surface expression identifies developing thymocytes which audition for T cell antigen receptor-mediated positive selection. *Int. Immunol.* **9**: 1139–50.

31. Sant'Angelo, D. B., Lucas, B., Waterbury, P.G., Cohn, B., Brabb, T., Goverman, J., Germain, R.N., and Janeway, C.A. (1998) A molecular map of T cell development. *Immunity.* **9**: 179–86.

32. Gumley, T., McKenzie, I & Sandrin, M. (1995) Sequence and structure of the mouse ThB gene. *Immunogenet.* **42**: 221–4.

33. Kappler, J.W, Roehm, N., Marrack, P. (1987) T cell tolerance by clonal elimination in the thymus. *Cell.* **49**: 273.

34. Chan, S.H, Benoist, C., Mathis, D. A. (1993) Challenge to the instructive model of positive selection. Immunol Rev. **135**: 119–31.

35. Henderson, S.C., Berezovskaya, A., English, A, Palliser D., Rock, K.L, and Bamezai, A. (1998) $CD4^+T$ cells mature in the absence of major histocomplatibility complex class I and class II expression in Ly-6A.2 transgenic mice. *J. Immunol.* **161**: 175–82.

36. Zerrahn, J., Held, W., Raulet, D. (1997) The MHC reactivity of the T cell repertoire prior to positive and negative selection. *Cell.* **88**: 627–36.

37. Merkenschlager, M., Graf, D., Lovatt, M., Bommhardt, U., Zamoyska, R., and Fisher, A.G. (1997) How many thymocytes audition for selection? *J. Exp. Med.* **186**: 1149–58.

38. Wang F., Huang C-U., and Kanagawa, O. (1998) Rapid deletion of rearranged T cell antigen receptor (TCR) Valpha-Jalpha segment by secondary rearrangement in the thymus: Role of continuous rearrangement of TCR-alpha chain gene and positive selection in the T cell repertoire formation. *Proc. Nat. Acad. Sci. USA..* **95**: 11834–39.

